# Therapeutic impact of purified *Trichoderma viride* l-asparaginase in murine model of liver cancer and in vitro Hep-G2 cell line

**DOI:** 10.1186/s43141-023-00493-x

**Published:** 2023-03-30

**Authors:** Dina H. El-Ghonemy, Sanaa A. Ali, Rehab M. Abdel-Megeed, Ali M. Elshafei

**Affiliations:** 1 Microbial Chemistry Department, Biotechnology Research Institute, National Research Centre, 33 El Buhouth St, Giza, EG-12622 Egypt; 2 Therapeutic Chemistry Department, Pharmaceutical and Drug Industries Research Institute, National Research Center, 33 El Buhouth St., Giza, EG-12622 Egypt

**Keywords:** *Trichoderma viride*, Glutaminase-free L-asparaginase, Anti-tumor agent, Purification, Cytotoxicity

## Abstract

**Background:**

Hepatocellular carcinoma (HCC) is among the common cancers, but difficult to diagnose and treat. l-asparaginase has been introduced in the treatment protocol of pediatric acute lymphoblastic leukemia (ALL) since the 1960s with a good outcome and increased survival rates to nearly 90%. Moreover, it has been found to have therapeutic potential in solid tumors. Production of glutaminase-free-l-asparaginase is of interest to avoid glutaminase-related toxicity and hypersensitivity. In the current study, an extracellular l-asparaginase that is free of l-glutaminase was purified from the culture filtrate of an endophytic fungus *Trichoderma viride*. The cytotoxic effect of the purified enzyme was evaluated in vitro against a panel of human tumor cell lines and in vivo against male Wister albino mice intraperitoneally injected with diethyl nitrosamine (200 mg/kg bw), followed by (after 2 weeks) oral administration of carbon tetrachloride (2 mL/kg bw). This dose was repeated for 2 months, and after that, the blood samples were collected to estimate hepatic and renal injury markers, lipid profiles, and oxidative stress parameters.

**Results:**

l-asparaginase was purified from *T. viride* culture filtrate with 36 purification folds, 688.1 U/mg specific activity, and 38.9% yield. The highest antiproliferative activity of the purified enzyme was observed against the hepatocellular carcinoma (Hep-G2) cell line, with an IC_50_ of 21.2 g/mL, which was higher than that observed for MCF-7 (IC_50_ 34.2 g/mL). Comparing the DENA-intoxicated group to the negative control group, it can be demonstrated that l-asparaginase adjusted the levels of the liver function enzymes and the hepatic injury markers that had previously changed with DENA intoxication. DENA causes kidney dysfunction and altered serum albumin and creatinine levels as well. Administration of l-asparaginase was found to improve the levels of the tested biomarkers including kidney and liver function tests. l-asparaginase treatment of the DENA-intoxicated group resulted in a significant improvement in the liver and kidney tissues to near normal similar to the healthy control group.

**Conclusion:**

The results suggest that this purified *T. viride* l-asparaginase may be able to delay the development of liver cancer and may be used as a potential candidate for future application in medicine as an anticancer medication.

## Background

Hepatocellular carcinoma (HCC) is one of the most common types of cancer and ranks the third among all cancer deaths, where nearly 7, 50,000 patients died annually from HCC [1, 2]. In Egypt, HCC accounts for 11.8% of all digestive organ tumors and 1.7% of all cancers [3]. Several risk factors for HCC have been determined, among which, the most important are infections with hepatitis B and C viruses and consumption of food contaminated with the fungal toxin aflatoxin (A & B_1_) [4–6]. Given the limited therapeutic efficacy in advanced HCC, it is essential to study new effective and less toxic therapeutic agents. L-asparagine (Asn) is a non-essential amino acid (AA) that can be taken up through diet or synthesized from central metabolic pathways by the enzyme asparagine synthetase (ASNS) from aspartic acid (Asp) and glutamine (Gln). l-asparaginase (L-ASNase) is an enzyme that hydrolyzes Asn into Asp and ammonia [7]. Systemic administration of bacterial L-ASNase has been reported to reduce the bioavailability of Asn as well as to eradicate rapidly proliferating cancer cells with a high demand for exogenous Asp [8]. Currently, it is a cornerstone drug for the treatment of the most common pediatric cancer, ALL. Because these lymphoblasts lack ASNS expression, their survival is dependent on extracellular Asn uptake. Interestingly, recent studies have demonstrated that L-ASNase has clinical potential for the treatment of other aggressive subtypes of hematological or solid cancers [9]. L-asparaginases are found in numerous organisms, such as plants, animals, and microorganisms. They are also found in the serum of rodents but not in humans. Microorganisms including bacteria and fungi were reported to be an effective and low-cost source of L-asparaginase, because it is difficult to extract the current enzyme from plants and animals [10]. L-asparaginases from *Escherichia coli* and *Erwinia chrysanthemi* have been used to treat leukemia, but with some evidence of toxicity induced by other enzymes like l-glutaminase and urease [11]. As a result, finding new sources of this enzyme that is free of l-glutaminase and do not have negative side effects is an important goal for researchers [12].

Submerged fermentation is the most widely used method for producing l-asparaginase worldwide (SmF). However, this method has a number of drawbacks, including high costs and a lack of product concentration. In addition, because of the large volume of effluent produced, a large amount of wastewater must be handled and disposed of during the subsequent processing [13]. Solid state fermentation (SSF) is a particularly efficient method, as it produces significantly more product than submerged fermentation. Furthermore, SSF has several advantages over SmF, including improved solid waste management, lower energy requirements, a lower risk of bacterial contamination, less waste water production, and simpler product extraction without the need for labor-intensive post-fermentation treatment procedures [14]. Agro-industrial byproducts were utilized in microbial enzyme production using SSF. Wheat bran is considered the universal substrate because it has a completely nutritious medium for different microorganisms and offers a large surface area as it remains loose under moist conditions [15].

Diethylnitrosamine (DENA) is frequently used to induce HCC in experimental animals [16, 17]. Earlier studies have demonstrated how liver function tests, such as alanine aminotransferase (ALT) and aspartate aminotransferase (AST) [17, 18], can reflect immune and nutritional status. They have been shown to be significant predictors of oxidative stress in a variety of diseases. Moreover, ALT and AST and other parameters of liver function tests including total and direct bilirubin and serum albumin could be used as reference indices for HCC diagnosis [19–21]. Therefore, the goal of the present study was to evaluate the cytotoxic effect of glutaminase-free-l-asparaginase purified from a terrestrial fungus *Trichoderma viride*, in vitro against the growth of different human tumor cell lines, and to investigate the preventive and therapeutic effects of the current enzyme against DENA-induced HCC in mice.

## Methods

### Chemicals

L-asparagine, glutamine, and diethyl nitrosamine (DENA) were bought from Sigma-Aldrich Chemicals Co. (St. Louis, MO, USA). Randox Company provided the kits needed to measure oxidative stress indicators and liver function (UK). All other chemicals are of the highest grade and are obtained from standard companies.

### Microorganism

*Trichoderma viride* used in the current work was obtained from culture collection stocks maintained by our Microbial Chemistry Department, Egypt. The fungus was cultured on potato dextrose agar slants (PDA) for 7 days at 28 °C and maintained at 4 °C.

### Experimental animal

Male Swiss albino mice weighing 25–30 g were obtained from the National Research Centre’s animal house in Egypt. The animals were housed in cages under standardized conditions of 26–28 °C temperature, 60% relative humidity, and a 12-h light/dark cycle. The animals were given unlimited access to water ad libitium and were fed a commercial pellet diet. All mice were properly cared for and handled in accordance with the National Research Centre’s institutional animal ethics committee. All animal care procedures strictly adhered to the National Institute of Health’s ethical procedures.

### L-asparaginase production under SSF and enzyme extraction

Erlenmeyer flasks (500 mL capacity) contain 10 g of rice husk (RH) and wheat bran (WB) in a 6.0:4.0 ratio moistened with synthetic medium (75%) consisting of (g/L) glucose 10, casein 15, NaNO_3_ 1.5, L-asparagine 1.0, MgCl_2_ 0.5, and Tween-20 3.0 mL and adjusted at pH 5.0 [14]. After sterilization (121 °C for 20 min), the flasks were cooled and inoculated with 2.0 mL of spore suspension of 1 × 10^8^ spores/mL, prepared from a 7-day-old culture by suspending the spores in sterile Tween-80 water (0.1%). All flasks were incubated at 28 °C for 4 days.

Under aseptic conditions, the crude enzyme was extracted from the moldy substrate by adding 100 mL of Tris–HCl buffer (0.05 M, pH 8.0) to each flask. The flasks were shaken for 1 h at room temperature on a rotary shaker (180 rpm). The slurry obtained was centrifuged for 15 min at 4 °C at 8000 rpm, and the developed clear supernatant was used for enzyme assay.

### Enzyme assay and protein estimation

l-asparaginase activity was determined using the method described by Nakahama et al. [22]. The reaction mixture contained 0.5 mL of clear supernatant, 0.5 mL of 0.04 M L-asparagine (L-glutamine was added as the substrate to determine l-glutaminase activity), and 0.5 mL of Tris–HCl buffer (0.05 M, pH 8.0) and was incubated at 37 °C for 30 min. After incubation time, 0.5 mL of 1.5 M trichloroacetic acid (TCA) was added to stop the reaction, and then, 0.1 mL of this mixture was transferred to a clean test tube containing 3.7 mL of distilled water and 0.2 mL of Nessler’s reagent and allowed to stand at room temperature for 10 min. The ammonia released was colorimetrically measured by reading the absorbance at 450 nm with a Cary-100 UV–Vis spectrophotometer (Agilent Technologies, Frankfurt, Germany). Under standard assay conditions, one unit (U) of enzyme activity was defined as the amount of enzyme that catalyzes the release of 1 μmole of NH_3_/min/mL. The protein content was estimated using the Bradford method [23].

### Enzyme purification

The crude enzyme filtrate was precipitated by gradually adding ethanol (− 20 °C) with stirring until 50% saturation was reached. The mixture was kept at 4 ºC for 12 h and then centrifuged at 12,000 × g for 20 min at 4 ºC. The precipitate obtained was dissolved in a small volume of 0.05 M Tris–HCl buffer (pH 8.0) and dialyzed for 24 h at 4 ºC against the same buffer (0.01 M, pH 8.0) and then tested for enzyme activity and protein concentration. The dialysate was loaded onto a Sephadex G-100 column (1.5 × 100 cm) pre-equilibrated with Tris–HCl buffer (0.05 M, pH 8.0), and protein was eluted with the same buffer at a flow rate of 20 mL/h. Five mL fractions were collected, and the protein content of each elute was measured using the UV absorbance technique (Cary-100 UV–Vis spectrophotometer, Agilent Technologies, Frankfurt, Germany). Fractions with high-enzyme activity were pooled, lyophilized, and chromatographed on a Sephadex G-200 column (2.5 × 50 cm) pre-equilibrated with Tris–HCl buffer (0.05 M, pH 8.0), and protein elution was carried out at a flow rate of 15 mL/h. Active fractions were pooled, lyophilized, and stored at − 20 ºC for further studies.

### Cytotoxic effect of the purified enzyme

#### Cell culture

The procedure was performed in a sterile biosafety class II laminar air flow cabinet. The culture was kept in a DMEM medium containing a 1% antibiotic–antimycotic mixture (10,000 U/mL of potassium penicillin, 10,000 μg/mL of streptomycin sulfate, and 25 μg/mL of amphotericin B), 1% L-glutamine, and 10% heat-inactivated fetal bovine serum. Doxorubicin and DMSO were used as a positive and negative control, respectively [24].

#### Cell viability assay

The 3-[4,5-dimethylthiazole-2-yl)-2,5-diphenyltetrazolium bromide (MTT) assay was utilized to evaluate the cytotoxicity of l-asparaginase against a panel of human tumor cell lines, including Hep-G2 (human hepatocellular carcinoma), MCF-7 (breast cancer), HCT-116 (colon cell line), and DMEM A-549 (human lung carcinoma) [25]. After 10 days of culturing, the cells (5 × 10^4^ Cells/Well) were seeded in a fresh complete growth medium in 96-well microtiter plastic plates at 37 ºC for 24 h in a water-jacketed CO_2_ incubator (under 5% CO_2_). Fresh medium (without serum) was incorporated, and cells were incubated alone (control) or with an enzyme to 100 μg/mL final concentration. After 24 h, the medium was aspirated, and 40 μL of MTT salt (2.5 mg/mL) was added to each well and was incubated for 4 h at 37 ºC with 5% CO_2_, then each well received 200 μL of sodium dodecyl sulfate (SDS) in deionized water (10%) to terminate the reaction and dissolve the crystals that had formed. A microplate multi-well reader was used to measure the absorbance at 595 nm with a reference wavelength of 690 nm. Cell viability was determined according to the mitochondrial-dependent transformation of yellow MTT into purple. The data were represented as the mean percentage of viable cells compared to the corresponding control cultures that had been solvent-treated. From the linear equation of the dose-dependent curve of each sample, the half maximum growth inhibitory concentration (IC_50_) values were determined.

### Antineoplastic activity of purified L-asparaginase

#### Experimental design

Mice were divided into 4 groups (10/each) for a 16-week study period, after 1 week of acclimatization. Group I: mice were given saline as a normal control group. Group II: HCC was induced in mice via IP injection of DENA in normal saline (200 mg/kg bw), and 2 weeks later, CCl_4_ was orally administered at 1:1 dilution in corn oil as a carcinogenic effect promoter. DENA and CCl_4_ injections were repeated after 1 month from the first DENA injection. Group III: mice were injected with l-asparaginase (25 U/mL) via the same route three times with a 24-h interval between each administration. Group IV: DENA and CCl_4_ were given to mice as in group II and simultaneously treated with enzymes as in group III.

#### Blood sampling and preparation of tissue

At the end of the experiment, the animals were weighed, and blood samples were taken from the sublingual vein. Sera were separated through centrifugation for 15 min at 4000 rpm and stored at − 20 °C for a subsequent biochemical parameter analysis. After that, mice were sacrificed via cervical dislocation. For histopathological examination, liver and kidney tissues were carefully separated and stored in 10% formaldehyde.

### Estimated parameters

#### Estimation of liver function enzymes

ALT and AST activities were measured spectrophotometrically using commercially available Randox Company kits [26]. Alkaline phosphatase levels were determined in serum samples from all groups tested [27]. Biodiagnostic Kits (Biodiagnostic Co., Upton-Upon-Seven, Worcestershire, UK) were used to measure total bilirubin [28]. The method described by Belfield and Goldberg [29] was used to calculate alkaline phosphatase (ALP).

#### Estimation of kidney function and antioxidants

Creatinine was measured using the method described in the manufactured Kit (Biodiagnostic Co., Upton-Upon-Seven Worcestershire, UK) [30]. Albumin was estimated as the manufacturer described Biodiagnostic Kits (Biodiagnostic Co., Upton-Upon-Seven, Worcestershire, UK) [31].

#### Determination of some antioxidants

The basic level of hydrogen peroxide in liver homogenates was determined using Biodiagnostic Kits (Biodiagnostic Co., Upton-Upon-Seven Worcestershire. UK). Lipid peroxide was determined spectrophotometrically at 520 and 535 nm, using 1,1,3,3-tetramethoxy propane as a standard [32]. Nitric oxide was measured using Biodiagnostic Kits (Biodiagnostic Co., Upton-Upon-Seven Worcestershire, UK) at 540 nm [33].

#### Lipid profile

Serum total lipids, triglyceride, and cholesterol concentrations were determined in serum samples using standard diagnostic kits according to the methods of Zollner and Kirsch [34], Fossati and Prencipe [35], and Richmond [36], respectively.

### Histopathological examination

Rats were sacrificed, and their kidneys and livers were stored in a neutral formalin solution (10%). The specimens undergo trimming, washing, and dehydration in ascending alcohol concentrations. Tissue samples were cleaned with xylene, then immersed in paraffin, and cut into 4-micron-thick sections before being stained with hematoxylin and eosin (H&E stain) and examined under a microscope [37].

### Statistical analysis

The statistical analysis was carried out with the Instat-3 computer program (GraphPad software Inc., San Diego, CA, USA). The SPSS 12 program’s one-way analysis of variance (ANOVA) followed by the post hoc test was employed to evaluate the differences between the means of various groups. The significance level was set at *p* < 0.05 by using Tukey ҆s test, and the data were presented as means ± S.E.M.

## Results

### Purification of L-asparaginase

Extracellular l-asparaginase produced from *T. viride* was purified using ethanol precipitation, dialysis, and gel filtration on Sephadex G-100, followed by loading the most active fractions on a Sephadex G-200 column. The specific activity of the enzyme and purity increased with each purification step, while total protein, total activity, and yield declined proportionally. Purification of crude l-asparaginase resulted in 1789 ± 14.7 U of enzyme activity free from L-glutaminase, and recovery yield of 38.9%, with an increased specific activity from 19.3 to 688.1 U/mg protein and purification fold of 35.7 (Table 1).

**Table 1:** Summary of steps employed in the purification of l-asparaginase from *Trichoderma viride* culture filtrate

Purification step	Total activity (U)	Total protein (mg)	Sp. activity (U/mg protein)	Recovery (%)	Purification fold
Crude extract	4597 ± 154	238 ± 11.3	19.3	100	1.0
Ethanol precipitation (50%)	2863 ± 58.2	31.4 ± 2.15	91.2	62.3	4.7
Dialysis	2798 ± 36.8	29.4 ± 2.13	95.2	60.9	4.9
Sephadex G-100	2148 ± 23.5	6.4 ± 0.17	335.6	46.7	17.4
Sephadex G-200	1789 ± 14.7	2.6 ± 0.12	688.1	38.9	35.7

Data is expressed as mean ± SD of triplicates

### Cytotoxic effect of L-asparaginase

Purified *T. viride* l-asparaginase demonstrated anti-proliferative activity against the growth of Hep-G2 (67% at 100 ppm) and MCF-7 (38% at 100 ppm), while it was not effective against Hct-116 and DMEM A-549 cell lines. As a result, the Hep-G2 and MCF-7 were chosen for further investigation. The incubation of Hep-G2 and MCF-7 with increased doses of the current enzyme led to a gradual inhibition of cell growth, as evidenced by the low IC_50_ values of 21.2 μg/mL and 34.2 µg/mL, respectively.

### Determination of antineoplastic activity of purified enzyme

#### Influence of L-asparaginase on liver function tests

**Fig. 1:**
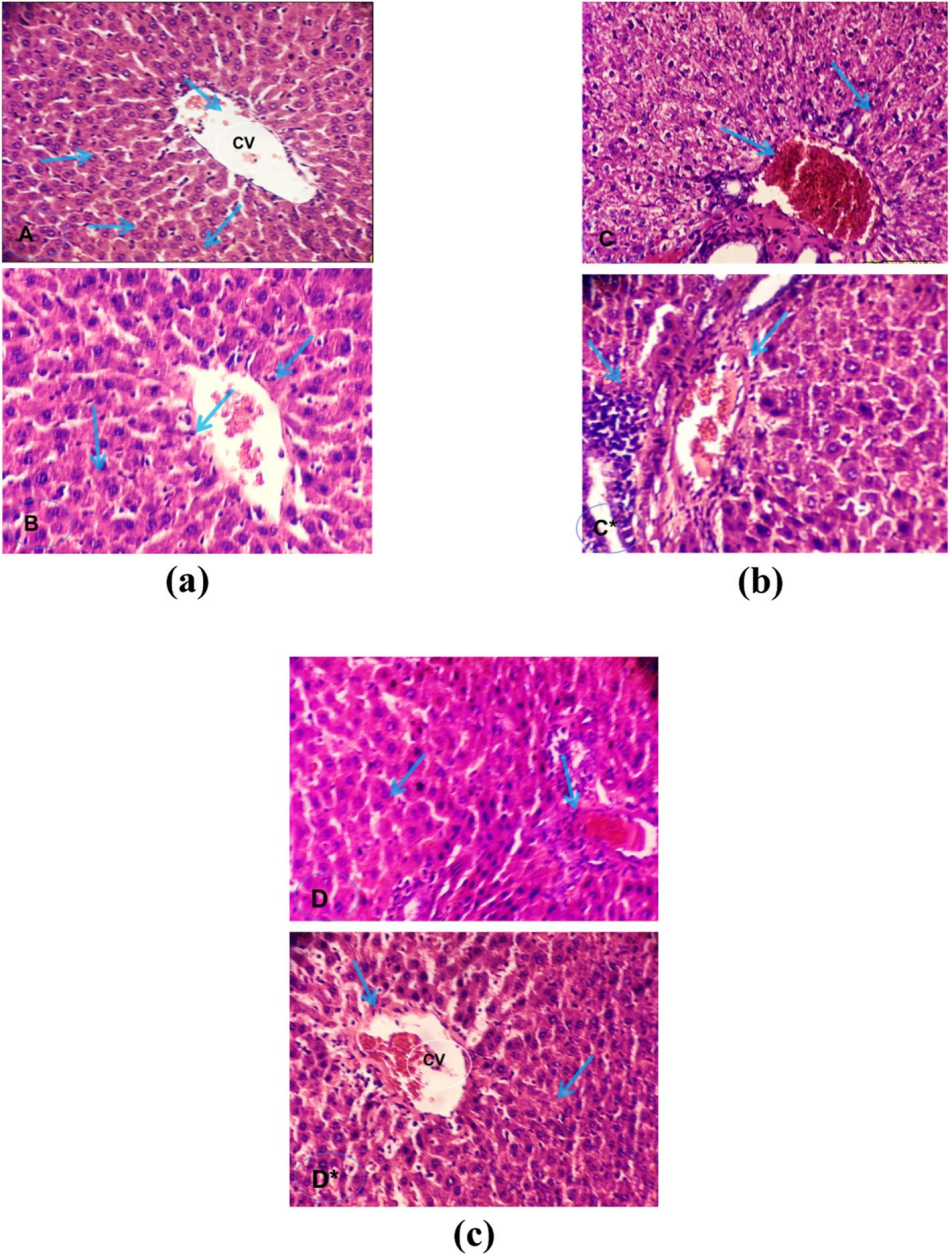
Liver section from **a** control rat group showed normal hepatocyte cells before (**A**) and after (**B**) l-asparaginase treatment (non-significant change with respect to control); **b** treated rat (C&C*) given diethyl nitrosamine DENA (chemotoxic agent) showed enlarged hyperchromatic nuclei with scattered mitosis, massive focal necrosis cirrhosis in hepatic parenchyma cells with inflammatory cells infiltration surrounding the dilated portal vein and sinusoidal dilatation and congestion; **c** cirrhotic rat treated l-asparaginase (D&D*) showed significant regression of hepatic fibrosis, reduced sinusoidal dilatation and congestion, restoration of most of the normal hepatocyte architecture with regular dark nuclei

The administration of the purified *T. viride* l-asparaginase to control animals does not result in a significant increase in total bilirubin, ALT, or AST. The ALP declared a slight elevation. Figure 1a revealed a non-significant difference when compared to the control hepatic group 200x. However, DENA intoxication significantly enhanced serum levels of AST, ALT, ALP, and total bilirubin, recording 173.83, 81.33, 207.1, and 1.69, respectively, when compared to the negative control group. The histopathological analysis shown in Fig. 1b revealed sinusoidal dilatation and congestion, as well as massive focal necrosis in the hepatic parenchyma. l-asparaginase treatment of the DENA-intoxicated group restored levels of liver function enzymes to near-normal levels (Table 2 and Fig. 1c). Moreover, hepatic fibrosis was significantly reduced in cirrhotic rats given L-asparaginase.

**Table 2:** Effect of *T. viride* l-asparaginase on liver function enzymes in serum of control and DENA-intoxicated

Parameter	Groups			
	Normal control (1)	Normal-treated enzyme (2)	(+ ve) DENA (3)	DENA-treated enzyme (4)
AST (U/mL)	116.2 ± 4.5^a^	117.5 ± 17.6^a^	173.83 ± 23.1^b^	117.5 ± 10.7^a^
ALT (U/mL)	55.2 ± 3.3^a^	54.7 ± 3.83^a^	81.33 ± 13.53^b^	61.33 ± 6.74^a^
ALP (U/L)	129.7 ± 4.1^a^	141.85 ± 35.7^b^	207.1 ± 7.5^c^	116.18 ± 12.8^a^
T. bilirubin (mg/dL)	1.34 ± 0.16^a^	1.48 ± 0.21^a^	1.69 ± 0.16^b^	1.53 ± 0.18^a^

Data are expressed as means ± SD of six mice in each group

Analysis of data is carried out by one-way (ANOVA) (analysis of variance) accompanied by post hoc (LSD, least significant difference) (CoStat, computer program)

Different letters indicate that these groups are significantly correlated at *P* > 0.05

Different letters are significantly different from each other while similar letters are not significantly different at *P* < 0.05

#### Effect of L-asparaginase on kidney function tests

**Fig. 2:**
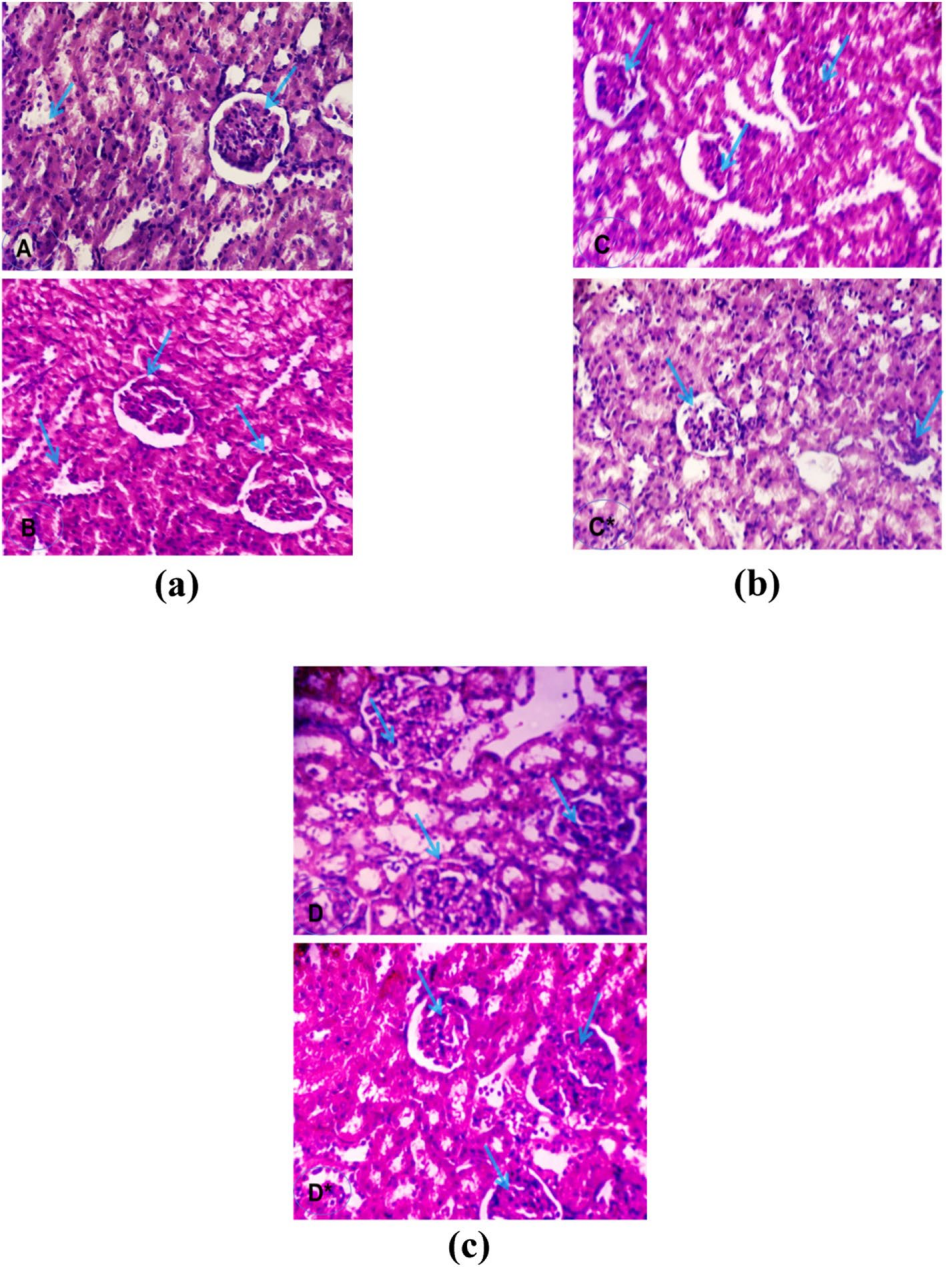
Kidney section from **a** control rat group showed normal histology kidney cells before (**A**) and after (**B**) l-asparaginase treatment (non-significant change with respect to control); **b** treated rat (C&C*) given diethyl nitrosamine DENA (chemotoxic agent) showed the appearance of the corpuscles with high cellularity and obliterated capsular space, proximal convoluted tubules with destructed epithelial lining of distal convoluted tubules, shrunken glomeruli indicating degeneration of nuclei and increased Bowman’s space; **c** treated l-asparaginase (D&D*) showed a reduction of congestion of the blood vessels, the renal cortex of the renal corpuscle with nearly normal glomerulus which restored shrunken glomeruli and Bowman’s space

Treatment of healthy control animals with l-asparaginase resulted in a slight reduction in albumen levels, but no significant difference in creatinine levels when compared to the negative control group. Kidney section treated with l-asparaginase showed no significant change from the control group (Fig. 2a). In the DENA intoxication group, albumin levels were significantly lower, while creatinine levels increased significantly, recording 4.56 and 2.36, respectively, when compared to the negative control group. Figure 2b showed the appearance of corpuscles with high cellularity and obliterated capsular space. l-asparaginase treatment of the DENA-intoxicated group improved albumin and creatinine levels (Table 3). Figure 2c showed a reduction in blood vessel congestion, renal cortex, and a nearly normal glomerulus after l-asparaginase treatment.

**Table 3:** Effect of *T. viride* l-asparaginase on albumin and creatinine levels in serum of control and DENA-intoxicated

Parameter	Groups			
	Normal control (1)	Normal-treated enzyme (2)	(+ ve) DENA (3)	DENA-treated enzyme (4)
Albumin (g/dL)	6.78 ± 1.7^a^	5.95 ± 1.4^b^	4.56 ± 0.79^c^	5.63 ± 0.84^b^
Creatinine	0.64 ± 0.097^a^	0.75 ± 0.10^a^	2.36 ± 0.24^b^	0.63 ± 0.081^a^

Data *are* expressed as means ± SD of six mice in each group

Analysis of data is carried out by one-way (ANOVA) (analysis of variance) accompanied by post hoc (LSD, least significant difference) (CoStat, computer program)

Different letters indicate that these groups are significantly correlated at *P* > 0.05

Different letters are significantly different from each other while similar letters are not significantly different at *P* < 0.05

#### Effect of L-asparaginase on lipid profile

The administration of purified l-asparaginase to control animals resulted in a significant reduction in triglyceride, cholesterol, and total lipids when compared to the healthy group. The DENA-intoxicated group had a significant increase in both triglyceride and total lipids, but the cholesterol value was non-significant relative to the negative control group. l-asparaginase treatment of the DENA-intoxicated group improved the altered percentage of triglyceride levels while having no significant effect on the level of cholesterol. A significant reduction in total lipid was observed near the normal in the treated enzyme group (Table 4).

**Table 4:** Effect of *T. viride* l-asparaginase on lipid profile in serum of control and DENA-intoxicated

Parameter	Groups			
	Normal control (1)	Normal-treated enzyme (2)	(+ ve) DENA (3)	DENA-treated enzyme (4)
Triglyceride	65.20 ± 3.74^a^	46.18 ± 6.29^b^	80.18 ± 8.45^c^	67.95 ± 8.17^a^
cholesterol	70.79 ± 2.54^a^	47.10 ± 3.75^b^	73.24 ± 16.58^a^	62.08 ± 5.77^a^
T. Lipids	313.5 ± 17.5^a^	260.0 ± 15.31^b^	377.67 ± 57.81^c^	359.44 ± 48.3^b^

Data are expressed as means ± SD of six mice in each group

Analysis of data is carried out by one way (ANOVA) (analysis of variance) accompanied by post hoc (LSD, least significant difference) (CoStat, computer program)

Different letters indicate that these groups are significantly correlated at *P* > 0.05

Different letters are significantly different from each other while similar letters are not significantly different at *P* < 0.05

#### Modulation effect of l-asparaginase on oxidative stress biomarkers

l-asparaginase treatment of healthy animals resulted in a slight increase in oxidative stress biomarkers, with 4.84 and 5.53 for lipid peroxide and Nox, respectively, when compared to the negative control group. In the DENA intoxication group, lipid peroxide and Nox values were significantly higher of 6.00 and 7.87, respectively, when compared to the negative control values (3.12 and 1.99). While treating the DENA-intoxicated group with the current enzyme, resulted in significant improvements of 4.69 and 6.45, respectively, and reduced to be near group 2 (control group that administrated l-asparaginase only) (Table 5).

**Table 5:** Effect of *T. viride* F2 l-asparaginase on lipid peroxide and nitric oxide levels in serum of control and DENA-intoxicated

Parameter	Groups			
	Normal control (1)	Normal-treated enzyme (2)	(+ ve) DENA (3)	DENA-treated enzyme (4)
Lipid peroxide	3.12 ± 0.36^a^	4.84 ± 1.13^b^	6.00 ± 0.84^c^	4.69 ± 0.47^b^
NOx	1.99 ± 0.12^a^	5.53 ± 2.01^b^	7.87 ± 1.89^c^	6.45 ± 1.37^b^

Data are expressed as means ± SD of six mice in each group

Analysis of data is carried out by one-way (ANOVA) (analysis of variance) accompanied by post hoc (LSD, least significant difference) (CoStat, computer program)

Different letters indicate that these groups are significantly correlated at *P* > 0.05

Different letters are significantly different from each other while similar letters are not significantly different at *P* < 0.05

## Discussion

An important aspect of today’s pharmaceutical industry is the production or processing of enzymes for use as drugs. Therapeutic enzymes are used for a wide range of purposes. Cancer treatment is a significant potential therapeutic application for enzymes. L-asparaginases have emerged as particularly promising enzymes for the treatment of acute lymphoblastic leukemia as well as in a variety of other tumor therapy clinical trials [38]. l-asparaginase deprives neoplasms of essential nutrients and causes selective death of asparagine-dependent tumor cells by depriving them of this amino acid [39]. As a result of the importance of L-asparaginases in the pharmaceutical and industrial sectors, the scientific community has been encouraged to look for new microbial isolates that produce L-asparaginases with novel properties, as well as to investigate different resources for the effective production of these enzymes [40]. Thus, the present study is focused on the isolation and purification of glutaminase-free l-asparaginase from *T. viride* culture filtrate. The purified l-asparaginase exhibited a specific activity of 688 U/mg protein, 38.9% recovery yield, and 36 purification folds, which was higher than the specific activities of L-asparaginases purified from *Mucor hiemalis* (69.43 U/mg) [41], and *Penicillium* sp (13.97 U/mg) [42]. In addition, this enzyme was purified 106-fold from *Pseudomonas aureginosa* 50,071 through precipitation by ammonium sulfate, followed by gel filtering on Sephadex G-100, and CM sephadex C-50 columns [43], while an intracellular l-asparaginase was isolated from *Streptomyces* sp. PDK2 with a final yield of 2.18% and up to 83-fold purification using gel filtration on Sephadex G-200 [44]. In addition, l-asparaginase was purified from *Bacillus* strain DKMBT10 in two steps, yielding 43% recovery [45]. Moreover, this enzyme was purified from *S. longsporusflavus* (F-15) and *S. albidoflavus* up to 30.5-fold with 19.1% recovery, and 99.3-fold with 20% recovery, respectively [46, 47].

Cytotoxicity of the current l-asparaginase against various human cancer cell lines was investigated, and the results revealed that the enzyme was toxic to HepG-2 (67%) with IC_50_ of 21.2 µg/mL, and MCF-7 (38%) with IC_50_ of 34.2 µg/mL, while it was not effective against Hct-116 and DMEM A-549. These findings agreed with those of other studies [48, 49]. Cappelletti et al. [50] investigated the cytotoxicity of a new l-asparaginase from the pathogenic strain *Helicobacter pylori* CCUG 17,874 in vitro. They claimed that the most affected were AGS and MKN-28 gastric epithelial cells. L-asparaginases were found to suppress the glycosylation of a variety of synthesized proteins [51]. According to El-Naggar et al. [52], these enzymes can disrupt colon cancer cells by altering the interactions between endothelial cell microvasculature and extracellular matrix components. Asparagine participates in the Krebs cycle after being transformed into oxaloacetic acid, influencing cell metabolism. Moreover, the intracellular asparagine aids in the uptake of extracellular serine that is necessary for the synthesis of nucleic acids [53]. While the deficiency of asparagine is likely to prevent cell growth because the proliferation of human leukemic cell lines as well as other tumor cells requires massive amounts of such amino acid to yield enough energy and synthesize biomolecules [54]. Tumor cells can not manufacture L-asparagine because they lack the L-asparagine synthetase gene, which causes them to starve and die. Additionally, the lack of asparagine causes the cell cycle to be disrupted [55].

Hepatotoxicity is commonly associated with hyperlipidemia [56], which is consistent with our findings, which show an increase in the serum levels of TC, TG, LDL-C, and VLDL-C while the level of HDL-C was significantly lower in the DENA/CCl_4_-treated group as compared to the control group. This was known as the DENA/CCl_4_ hyperlipidemic effect, which was caused by an increase in free radicals released as a result of lipid peroxidation in biological membranes and tissues, impairing liver functions and being a major cause of hormonal imbalance. This imbalance caused lipid leakage into circulation and, as a result, hyperlipidemia via its multiple effects on lipid metabolism, including improved synthesis of TC, TG, and LDL-C [57]. Moreover, elevated triglyceride levels can be attributed to an imbalance between both the rate of production and the rate of release of triglycerides into the systemic circulation by parenchymal cells [58]. After following a high-fat diet for 11 weeks, the serum levels of TC, TG, LDL-C, and VLDL-C were significantly higher in the obese group as compared to the control group, while the level of HDL-C was non-significantly decreased. This is consistent with previous experimental findings and clinical observations, which have helped to clarify the role of fatty acids in altering the metabolic processes of the liver, where fat accumulation encourages the liver to synthesize more triglycerides while failing to export them. As a result, lipid metabolism, fatty acid biosynthesis, and lipoprotein formation are affected [59, 60]. According to the results of the current study, DENA/CCl_4_ administration to rats resulted in induced hepatotoxicity by significantly raising ALT, AST, and ALP activities as well as serum levels of total and direct bilirubin and significantly lowering albumin concentration when compared to the control group. The higher levels of liver enzymes observed in Swiss albino mice injected with DEN/CCl_4_ may be the result of DNA damage and subsequent cell degeneration brought on by alkylating DNA structures. This could lead to leakage from damaged liver tissues, increased production and leakage in circulation, and decreased hepatic clearance [61, 62]. Our findings revealed that adding a high-fat diet to obesity caused a non-significant change in serum levels of ALT, ALP, total and direct bilirubin, and albumin concentration, but a substantial rise in AST when compared to the control. This could be attributed to the fact that an 11-week high-fat diet was insufficient to induce severe liver damage, whereas abnormalities in the levels of ALT and ALP in serum occur only in severe cases of advanced nonalcoholic fatty liver disease [63].

In the present investigation, obese rats were given a diet high in fat, and subsequently, DENA and CCl_4_ treatments were administered. In contrast to the obese group, the AST activity and albumin concentration were significantly lower, while the serum levels of total and direct bilirubin, ALT and ALP activities, and total and direct bilirubin levels were all significantly higher. These results were in line with earlier studies showing that DENA induces hepatic inflammation and elevates blood-liver enzymes in obese rats within a few days of dosing [64, 65]. Reactive oxygen species (ROS) and oxidative stress play a substantial role in DENA-induced hepatotoxicity [66, 67]. In this investigation, DENA/CCl_4_ treatment significantly reduced the hepatic activities of SOD, CAT, and GSH in comparison to the control group while significantly raising the level of hepatic MDA. This resulted from a rise in the intracellular alkylating metabolites of DENA/CCl_4_, which led to oxidative stress, lipid peroxidation, and excessive free radical production [68]. This is also in line with earlier studies showing that DENA impairs sophisticated antioxidant defense mechanisms by increasing the formation of reactive oxygen species and membrane lipid peroxidation, both of which lead to bio-membrane damage. Our results are consistent with those of Helal et al. [69], who discovered that injection of DENA/CCl_4_ significantly increased hepatic MDA levels while lowering hepatic SOD and CAT activity as well as GSH levels [69]. The levels of MDA, SOD, or catalase activity were unaffected by the oral supplementation of a high-fat diet, while the levels of GSH in the livers of obese rats fell noticeably in comparison to the rats in the control group. The absence of an increase in aminotransferases in the blood was consistent with non-hepatocyte damage [70]. Hepatic MDA, CAT, and GSH levels were unaffected by DENA/CCl_4_/obese treatment, but hepatic SOD levels were significantly lower when compared to obese rats [61]. A high-fat diet may delay the production of toxic carcinogenic agents in the liver [71]. According to Goldfarb et al. [72], polyunsaturated fatty acid-rich fish oil reduces post-operative metastasis and increases recurrence-free survival in melanoma-bearing rodents. Furthermore, mice fed a high-fat diet and injected with DENA had lower tumor burdens than mice fed a normal diet [73].

According to liver histopathology in rats, DENA/CCl_4_ treatment induced lymphocytic infiltrate, fibroblastic proliferation in a portal area with newly formed bile ductules, as well as in intralobular with a tendency to form nodules and acini. Individualization and dysplastic alterations of hepatocytes with lytic necrosis and hemorrhage were also observed in the liver, as was dilatation of hepatic sinusoids. All of these histopathological changes can be attributed to an increase in free radicals caused by the formation of MDA and 4-hydroxynonenalmustard, the primary alkylating metabolites of DENA produced in the liver that attack cellular targets such as DENA [74]. Elaidy et al. [75] observed a disturbed architecture of thick plates, nests, and sheets of hepatocytes with enlarged nuclei, moderate pleomorphism, and few apoptotic and necrotic cells in the DENA/CCl_4_-control group. Histopathological examination of hepatic sections from rats fed a high-fat diet revealed lymphocyte-filled hepatic sinusoids, as well as intralocular fibroblastic proliferation forming septa [75]. There was also periportal hepatic necrosis and neutrophil recruitment. This is consistent with the findings of Hussein et al. [76], who discovered the lobular activity, portal inflammation, and an increase in fat droplet accumulation in hepatic tissues in mice fed a high-fat diet for 8 weeks compared to control rats. Furthermore, cross-sectioned hepatic tissue from the DENA/CCl_4_/obese group showed hepatocyte dysplasia, necrosis, and typia, as well as intralobular fibroblastic proliferation. Besides, there is a portal area with intense lymphocytic infiltrates and biliary epithelial hyperplasia in the liver [77]. This is most likely due to DENA, which increases the lipotoxicity of a high-fat diet and accelerates the development of fibrosis and cirrhosis [77]. Our findings are consistent with the findings of Darvin et al. [78], who discovered multifocal mononuclear cell infiltration, congestion, and hydropic hepatocyte degradation in rat liver sections treated with DENA and CCL_4_.

## Conclusion

Glutaminase-free l-asparaginase purified from *Trichoderma viride* culture filtrate, with 36 purification folds, 688.1 U/mg specific activity, and 38.9% yield, has been found to exert statistically significant high antiproliferative activity against hepatocellular carcinoma both in vitro using Hep-G2 cell line, with an IC_50_ of 21.2 g/mL, and in vivo using DENA intoxicated murine model, where the latter showed a significant resolution of the hepatocellular damage approaching the healthy control group. Therefore, *T. viride* l-asparaginase represents a promising therapeutic agent in hepatocellular carcinoma.

## Data Availability

All data generated or analyzed during this study are included in this article.
